# Impact of Nitric Oxide Metabolites On Early Perioperative Biochemical Changes and Postoperative Complications in High-BMI Patients Undergoing On-Pump CABG: an Exploratory Observational Cohort Study

**DOI:** 10.1007/s10557-026-07903-5

**Published:** 2026-06-27

**Authors:** Regina Samborska, Włodzimierz Płotek, Karolina Iwona Kulinska, Katarzyna Czerniak, Alina Kiełpińska, Krzysztof Kusza, Joanna Pielok, Maria Wołuń-Cholewa, Hanna Billert

**Affiliations:** 1 Department of Cardiac Surgery, Józef Struś Multi-Specialty Municipal Hospital, Szwajcarska 3, Poznań, 61-285 Poland; 2 Clinic of Anaesthesiology and Intensive Therapy, University Clinical Hospital, No 4, Dr Kazimierza Jaczewskiego 8, Lublin, 20-954 Poland; 3 Chair and Department of Cell Biology, Poznan University of Medical Sciences, Rokietnicka 5D, Poznań, 60-806 Poland; 4 Department of Gene Expression, Institute of Molecular Biology and Biotechnology, Faculty of Biology, Adam Mickiewicz University, Uniwersytetu Poznańskiego 6, Poznań, 61- 614 Poland; 5 Center for Advanced Technologies, Adam Mickiewicz University, Uniwersytetu Poznańskiego 10, Poznań, 61-614 Poland; 6 Department of Anaesthesiology, Intensive Therapy and Pain Management, Poznan University of Medical Sciences, Grunwaldzka 55, Poznań, 60-352 Poland; 7 Department of Anaesthesiology and Intensive Therapy Teaching, Poznan University of Medical Sciences, Święcickiego 6, Poznań, 60-781 Poland

**Keywords:** Nitrites, Nitric oxide, Cardiopulmonary bypass, Obesity, Overweight, Inflammation, Perioperative care

## Abstract

**Purpose:**

Perioperative metabolic and inflammatory changes in patients with elevated body mass index (BMI) undergoing cardiac surgery are critical but incompletely understood, despite their impact on postoperative outcomes. Nitric oxide (NO) metabolites, particularly nitrites and nitrates, are potential early markers of kidney injury and early postoperative inflammatory marker response. This study evaluates perioperative changes in the plasma nitrite/nitrate ratio, selected biochemical variables and intraoperative haemodynamic parameters in overweight and obese patients undergoing on-pump coronary artery bypass grafting (CABG), and examines their association with early postoperative complications.

**Methods:**

In the exploratory observational cohort study, overweight and obese adult patients undergoing on-pump CABG with standard anaesthesia were followed using serial perioperative blood sampling at six perioperative time points (T1–T6), including periods before and after cardiopulmonary bypass (CPB) and the early postoperative phase. Assessed parameters included blood gases, osmolality, NO metabolites, lactate, glucose, creatinine, and inflammatory markers: CRP, leukocyte count. Mean arterial pressure (MAP), systolic blood pressure (SBP), diastolic blood pressure (DBP), and heart rate (HR) were analysed to provide clinical context.

**Results:**

Overweight and obese patients showed no significant differences in baseline or perioperative characteristics. The nitrite/nitrate ratio increased significantly before CPB compared with pre-anaesthesia values (T2 vs. T1, *p* < 0.001) and remained elevated 10 min after aortic cross-clamping (AoX) (T3 vs. T1, *p* < 0.01), followed by rapid normalization after clamp release. Significant perioperative changes were also observed in pH, pO₂ and pCO₂, with increased lactate and glucose concentrations and decreased osmolality 10 min after AoX (T3 vs. T1, *p* < 0.001). These shifts coincided with reduced MAP, SBP, and DBP, and a delayed HR increase (all time points vs. T1, *p* < 0.001). Exploratory tree-based analyses identified the nitrite/nitrate ratio and aortic cross-clamp duration as variables associated with early postoperative creatinine and CRP levels, respectively.

**Conclusion:**

The plasma nitrite/nitrate ratio and AoX duration are associated with early postoperative creatinine increase and inflammatory marker response, respectively, in overweight and obese patients undergoing on-pump CABG.

## Background

The prevalence of overweight and obesity in patients is increasing every year around the world. According to a WHO report, in 2020, 39 million children under the age of 5 and more than 1.9 billion adults over the age of 5 will be overweight or obese [1]. Consequently, the number of surgical procedures performed on these patients also increases. Increased body weight and changes in body composition may significantly affect the perioperative inflammatory, biochemical, and NO-related metabolic responses, as well as the pharmacokinetic parameters of anaesthetics, including volume of distribution, clearance, and half-life, particularly during the elimination phase. A high BMI is often associated with ventilatory dysfunction and rapid oxygen desaturation. Furthermore, the presence of associated diseases in obese patients, such as obstructive sleep apnoea, may also limit the therapeutic dynamic range of anaesthetic agents [2].

In this context, perioperative management remains particularly challenging in this patient group [3]. Medical treatment of patients with elevated BMI is a challenge for cardiac surgeries, especially in procedures involving aortic cross-clamp (AoX), such as coronary artery bypass grafting (CABG). CABG is the gold standard in the surgical treatment of coronary artery disease (CAD), the most common post-pandemic cardiac pathology today [4]. Nevertheless, the procedure induces a serious complex inflammatory response due to blood exposure to the cardiopulmonary bypass (CPB), anaesthetic regimens, iatrogenic ischaemia-reperfusion (I/R) and surgical trauma. Release of the AoX causes acute reperfusion with anticoagulated blood at a high partial pressure of oxygen (PO_2_). Studies suggest that nitric oxide (NO)-related mechanisms are essential for predicting and preventing life-threatening complications [5, 6]. The mutual metabolic axis involving anaesthetics, mitochondrial activity, and exposure to oxygen deprivation and excess over short periods, leading to the production of NO-related metabolites, warrants further studies. Large randomized evidence has not demonstrated a clear difference between volatile and total intravenous anaesthesia in terms of major postoperative outcomes in cardiac surgery. Therefore, rather than comparing anaesthetic efficacy per se, the present study focused on perioperative NO-related metabolic changes and their potential association with early postoperative complications [7]. Furthermore, the interaction between NO metabolism, and perioperative physiological stress remains insufficiently understood. Limited data are available on their impact on nitric oxide-related metabolism, particularly in overweight and obese patients. Evaluating the metabolic, biochemical and hemodynamic parameters in the perioperative period of CABG in patients with a high BMI is crucial, particularly given the rising prevalence of overweight and obese individuals in the general population. Obesity is also linked to chronic adipose tissue hypoxia, which results in NO-dependent vascular and cellular dysfunction [8]. Exposure to high-level hyperoxia (exceeding 185 mmHg) during CPB, although inevitable in most cardiac procedures, is a high-risk factor for glycocalyx disruption and the e.q., postoperative kidney complications. The glycocalyx is also considered a mechanotransducer responsible for vascular NO release [8]. NO is subsequently metabolized into nitrites and nitrates, and the nitrite-to-nitrate ratio has been used as a safety indicator of NO production. Studies have shown that nitrite levels (not nitrates) reflect local iNOS activity during inflammation and are less influenced by diet [9, 10]. In addition, the levels of nitrites or the nitrite-to-nitrate ratio in biological samples have already been evaluated for their potential in clinical diagnostics [6]. In current clinical practice, mixed anaesthetic techniques are frequently used, which may further complicate the interpretation of perioperative biochemical responses. The mutual relationships among the factors: overweight/obesity, anaesthetics, hyperoxia during the CPB, and finally, altered metabolism of NO (indicating the intensity of the perioperative inflammatory reaction), create together the net of connections worth a more thorough evaluation.

This study aims to assess perioperative changes in the plasma nitrite/nitrate ratio, selected biochemical variables and haemodynamic parameters in patients with an elevated BMI subjected to the elective CABG procedures with particular attention towards the axis: overweight/obesity, gasometrical balance, and NO metabolites induced by inflammatory reaction assessed by C-reactive protein (CRP) and leucocyte count and to explore their association with early postoperative renal and inflammatory complications. We hypothesised that, distinct perioperative patterns of nitric oxide metabolism are related to physiological responses in patients with elevated BMI. Existing evidence addressing this issue in overweight and obese patients undergoing CABG remains scarce.

## Methods

This exploratory observational cohort study was conducted on adult patients with elevated BMI (≥ 25) undergoing elective isolated on-pump coronary artery bypass grafting between February 2013 and August 2013 at the Department of Cardiac Surgery, Józef Struś Multi-Specialty Municipal Hospital, Poznan, Poland. The study was approved by the Bioethics Commission of the Poznan University of Medical Sciences (No 124/13) and conducted according to the Declaration of Helsinki. Patients who were qualified and prepared for scheduled CABG surgeries with extracorporeal circulation were invited to participate in the research. Finally, 32 patients were qualified and classified according to BMI as overweight (25.0–29.9 kg/m²) or obese (≥ 30.0 kg/m²). The exclusion criteria were: BMI < 25, age < 18 or > 70 years; anti-inflammatory and/or immunosuppressive therapies; diagnosis of malignancy, chronic kidney disease (creatinine level > 2 mg/dL), liver disease (total bilirubin concentration in blood serum < 0.3 mg/dL or > 5 mg/dL), extrapulmonary infection (of the operative wound or any other infection); severe heart failure (New York Heart Association (NYHA) class IV); ejection fraction < 40%; psychiatric illness that may affect the ability to express formal consent and follow recommendations; alcohol addiction, unregulated diabetes; pre-existing coagulation disorders; bacterial infections and autoimmune diseases (C-reactive protein > 100 mg/L, leukocytosis (White Blood Cells > 12,000 cells/mm3), or body temperature > 37 °C.

### The Perioperative Anaesthesiological Procedure

The choice of maintenance anaesthesia (propofol-based or sevoflurane-based) was not protocol-driven and was made by the attending anaesthesiologist in accordance with standard clinical practice. One day before the operation, the patients received 2 mg estazolam orally, and 60 min before the surgery, they were premedicated with oral midazolam at a dose of 0.05–0.15 mg/kg. Cardiovascular medication was continued up to the day of the surgery; all patients received statins. Anaesthesia was induced with fentanyl administered at a dose of 3–7 µg/kg and etomidate at a dose of 0.2–0.4 mg/kg i.v. Once the bispectral index (BIS) values reached 40–45, rocuronium was administered intravenously at a dose of 0.6–0.8 mg/kg, and the patients were intubated. Volume-controlled intermittent positive pressure ventilation (IPPV) with TV = 6 mL/kg of ideal body mass, PEEP = 3–5 mmHg and F_I_O_2_ = 0.5 was provided by a Primus (Dräger, Germany) ventilator. All patients received a continuous i.v. infusion of fentanyl (0.02–0.05 µg/kg per·min). Anaesthesia was further maintained with either propofol: 60–100 µg/kg per·min, i.v. infusion or sevoflurane: 0.9–1.2 age-adjusted minimum anaesthetic concentration (MAC) before AoX and after declamping, according to group assignment. Anaesthesia depth was monitored using the bispectral index (BIS), with a target range of 40–55. The following parameters were monitored: continuous 2-lead ECG (II and V5), central venous pressure, direct arterial pressure measurement, pulse oximetry, capnography, central temperature (Philips IntelliVue), and hourly urine output.

## Cardiopulmonary Bypass (CPB) Procedure

Before CPB, heparin was administered intravenously at a dose of 400 IU/kg to achieve appropriate anticoagulation and prolong the activated whole-blood clotting time (ACT) to > 480 s, and then checked every 30 min. After weaning from CPB, heparinization was reversed by i.v. protamin sulfate 1 mg/100 IU heparin. After AoX 700-1,200 mL of cold cardioplegia solution (Cardioplegische Perfusion, Fresenius, Kabi) was injected in an antegrade fashion through the aortic bulb. Before CPB, the circuit was primed with 1,000 mL of balanced crystalloid solution, 100 mL of 20% mannitol, 60 mL of 5% sodium bicarbonate, and 8,000 IU of sodium heparin. During CPB, the mean arterial pressure (MAP) was maintained at 60–75 mmHg.

After the surgery, the patients were transferred to the postoperative intensive care unit (PICU). Pain management was individualised following an assessment with the 5-degree Prince Henry Hospital Pain Score (‘0’- no pain at cough, ‘4’- severe pain at rest). Pain control was provided by continuous infusion of morphine at a dose of 0.01–0.05 mg/kg per·hour i.v. and ketoprofen (Ketonal, Sandoz) at a dose of 100 mg/100 mL 0.9% NaCl i.v. every 8 h. The patients were extubated once they met standard criteria: efficient spontaneous breathing, responding to verbal commands, adequate muscle strength, stable blood pressure, normothermia, stable electrolyte and glucose levels, and no risk of excessive postoperative bleeding (defined as postoperative drainage over 100 mL per hour).

## Perioperative Blood Sampling and Study Endpoints T1-T6

Blood samples (5 mL) were drawn from the radial artery cannula into EDTA Monovette tubes (Sarstedt) with 6.5 mM N-ethylmaleimide (NEM, Sigma) added to block sulfhydryl groups and to avoid S-transnitrosylation at the following study endpoints: before the induction of anaesthesia (T1), before CPB (T2), 10 min after AoX (T3), 10 min after clamp release (T4), 30 min after weaning from CPB (T5), and on admission to the PICU (T6). Immediately after sampling, in the operating room, blood was centrifuged (4.500 rpm, 10 min) and plasma aliquots were stored at − 80 °C until biochemical measurements.

## Measurements

### Nitrite and Nitrate Concentrations

The plasma nitrite and nitrate levels were measured by ozone-based chemiluminescence (CL, Sievers^®^ Nitric Oxide Analyzer NOA 280i, GE Analytical Instruments, USA). CL signal is emitted from an activated state of nitrogen dioxide (NO_2_^*^) produced in the reaction of ozone with NO produced by acidic reduction of nitrate or nitrite. The detected energy was multiplied by a photomultiplier tube. Nitrite and nitrate concentrations were determined from their respective calibration curves. The detection limit concentration for measuring NO and its reaction products in liquid samples is 1 pM [11, 12]. For statistical analyses, the ratio of nitrite to nitrate concentrations was calculated at specific perioperative time points.

### Routine Biochemical and Biophysical Tests

Blood gas analysis, lactate concentrations, and WBC count were measured with a Cobas b221 system (Roche, Switzerland). Haemoglobin and haematocrit were measured spectrophotometrically with a sensitivity of approximately 3% using XS-1000i ™ Automated Hematology Analyzer (Sysmex, Japan). Glucose was measured with an enzymatic-colorimetric method with a sensitivity of 0.056 mmol/L (1 mg/dL). The CRP was determined using synthetic particles coated with antibodies against CRP with a sensitivity of 0.5 mg/L. The cTnI concentration was determined by a homogeneous chemiluminescent test method with a sensitivity of 17 ng/L. The creatine level was measured using the kinetic bichromatic colorimetric method with a sensitivity of 4 µmol/L. All measurements were done using the DIMENSION EXL 200 biochemical analyser (Siemens, Germany).

Basic indices of systemic inflammatory response, WBC count and CRP systemic concentrations, as well as indices of cardiac and renal injury, cardiac troponin (cTnI) and creatinine, were measured preoperatively, one day before the surgery and 6 h after surgery. Early postoperative renal marker was assessed using serum creatinine concentration measured 6 h after surgery. This variable was used as an early postoperative marker of renal dysfunction rather than as a definitive diagnosis of acute kidney injury (AKI), because the available dataset did not include complete 48-hour creatinine kinetics or urine-output-based AKI staging (Kidney Disease: Improving Global Outcomes guidelines). Early postoperative inflammatory response was assessed using CRP concentration and leukocyte count measured 6 h after surgery. These markers were interpreted as routine biochemical indicators of early inflammation rather than as a complete clinical definition of systemic inflammatory response syndrome.

## Intraoperative Haemodynamic Monitoring

Intraoperative haemodynamic parameters were assessed at predefined perioperative time points corresponding to blood sampling (T1-T6). The analysed haemodynamic variables included mean arterial pressure (MAP), systolic blood pressure (SBP), diastolic blood pressure (DBP), and heart rate (HR). Arterial blood pressure was measured invasively via an arterial catheter, and HR was continuously monitored using electrocardiography.

At T3 and T4, corresponding to the period of ongoing cardiopulmonary bypass, pulsatile haemodynamic variables such as SBP and DBP were physiologically altered and therefore not directly comparable with measurements obtained before and after CPB. Accordingly, SBP and DBP were analysed only at time points outside extracorporeal circulation (T1, T2, T5, and T6). Similarly, HR values were interpreted only at clinically relevant perioperative time points, whereas MAP was retained as the principal perfusion-related haemodynamic parameter across the entire perioperative period, including CPB.

Changes in haemodynamic parameters over time were analysed using repeated-measures ANOVA with Dunnett’s post hoc test, with T1 as the reference time point.

### Statistical Analysis

Sample size estimation was performed using G*Power 3.1.9.2 (University of Düsseldorf, Germany) for a repeated-measures ANOVA with one within-subject factor (time; six measurements: T1–T6). The calculation was based on the primary perioperative variable, namely the plasma nitrite/nitrate ratio. The assumed effect size was selected to reflect a clinically meaningful within-subject perioperative change in the nitrite/nitrate ratio across the predefined study time points. Assuming a moderate effect size (Cohen’s f = 0.33), α = 0.05, and statistical power of 80%, the minimum required sample size was estimated at 20 patients. To account for potential missing perioperative measurements and to improve the robustness of the longitudinal analysis, the final study group was increased to 32 patients.

Categorical variables were compared using the Chi-square test, whereas continuous variables were analysed using the Mann–Whitney U test. Data distribution was assessed before analysis. Variables without normality assumptions were log-transformed before repeated-measures ANOVA with Dunnett’s post hoc test. Sphericity was assessed using Mauchly’s test. Correlations were analysed using Spearman’s rank correlation coefficient. Statistical significance was accepted at *p* < 0.05 (Statistica ver. 13.3 software, TIBCO Software Inc., 2017). To reduce the risk of type I error associated with multiple testing, p-values were additionally adjusted using the Holm – Bonferroni procedure where appropriate. This correction was applied to the interpretation of repeated-measures ANOVA post hoc comparisons and correlation analyses, and did not alter the significance of the main results.

Decision tree analysis (CART methodology) was used as an exploratory approach to identify variables potentially associated with early postoperative complications, rather than to construct a definitive predictive model. A regression tree was constructed to model serum creatinine concentration at 6 h postoperatively as a marker of acute kidney injury. For each numerical feature, the absolute values of the correlation coefficients (|r|) were calculated to assess the strength of association regardless of direction. Based on these values, features with the highest absolute correlations were selected for further analysis. Predictor variables included nitrite/nitrate ratio (T2–T4), osmolality, glucose and lactate levels at T3, cardiopulmonary bypass time, AoX time, reperfusion time, pH and mean arterial pressure at T3, and BMI. Additionally, a classification tree was developed to predict the occurrence of an intensified inflammatory response defined as CRP > 100 mg/L. The same predictor set was used, with the addition of postoperative troponin and leukocyte count. Both models were limited to a maximum tree depth of 4 and validated using cross-validation.

All raw and processed data collected during perioperative timepoints (T1–T6) are available at MostWiedzy (DOI: [https://doi.org/10.34808/5b61-0w08]), under CC BY licence.

## Results

### Study Group Description

The data collected from the study group are summarised and presented in Table 1. It can be seen no statistically significant differences in demographic and clinical parameters such as age, gender, presence of diseases (myocardial infarction, hypertension, chronic obstructive pulmonary disease), smoking habits, cardiovascular capacity, and perioperative data among overweight and obese patients (calculated p-values for chi-squared and Mann-Whitney U test are above 0.05). However, it was observed that overweight patients had a statistically significantly longer hospital stay than obese patients (*p* = 0.04).

**Table 1:** Patient characteristics and perioperative data

	Patients			Overweight			Obesity		*P*
Age (years)*	60 (49–67)		60 (49–67)			59 (51–66)		0.84	
Men vs. women (n)	26 vs. 6		16 vs. 4			10 vs. 2		0.81	
BMI (kg/m^2^)*	29.2 (25.2–42)		27 (25.2–29.7)			31.35 (30.1–42)		-	
Myocardial infarction (no vs. yes)	17 vs. 15		9 vs. 11			8 vs. 4		0.23	
Hypertension (no vs. yes)	6 vs. 26		4 vs. 16			2 vs. 10		0.81	
COPD (no vs. yes)	3 vs. 29		1 vs. 19			2 vs. 10		0.27	
Smoking (no vs. yes)	25 vs. 7		15 vs. 5			10 vs. 2		0.58	
*Cardiovascular capacity*									
NYHA class I/II/III (n)	3/24/5	1/15/4			2/9/1			0.42	
CCS scale 1/2/3/4 (n)	3/20/8/1		3/11/5/1			0/9/3/0		-	
EuroSCORE II*	0.72 (0.50–8.05)		0.79 (0.50–8.05)			0.66 (0.55–2.02)		0.43	
EF (%) * *Perioperative data*	53.5 (40–65)		54 (42–65)			52.5 (40–58)		0.22	
Propofol vs. sevoflurane (n)	13 vs. 19		9 vs. 11			4 vs. 8		0.51	
Surgery duration (min)*	185 (130–220)		180 (130–220)			195 (135–220)		0.45	
CPB time (min)*	64.5 (43–101)		67 (43–96)			62 (46–101)		0.61	
AoX time (min)*	33.5 (21–51)		33.5 (21–48)			35 (24–51)		0.41	
Reperfusion time (min)*	23.5 (9–50)		24.5 (11–50)			23.5 (9–43)		0.91	
Number of grafts (n)	3 (2–4)		3 (2–4)			3 (2–4)		0.86	
Hospital stay (days)	8 (6–17)		8 (7–17)			8 (6–11)		0.04	

Categorical variables were compared using the Chi-square test; continuous variables were compared using the Mann–Whitney U test

*BMI * Body Mass Index, *COPD * Chronic Obstructive Pulmonary Disease, *NYHA *The New York Heart Association functional classification system, *CCS * Canadian Cardiovascular Society grading of angina pectoris, *EF * ejection fraction, *CPB * cardiopulmonary bypass, *AoX * aortic cross-clamp

*Median (min-max)

### Analysis of Biochemical Parameters Revealing Early Perioperative Changes Prior to Bypass Surgery (Time Point T2)

**Fig. 1:**
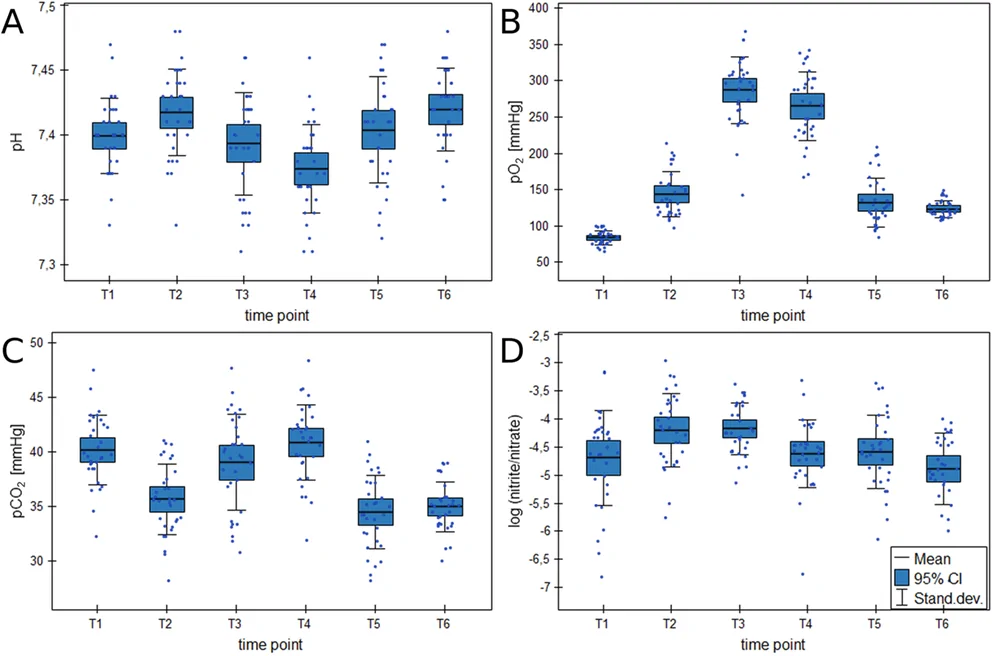
Analysis of biochemical parameters revealing early changes before cardiopulmonary bypass (CPB) (time point T2).Data are shown as mean values with 95% confidence intervals (95% CI) and standard deviation (Stand. dev.). pO_2_ - partial pressure of oxygen; pCO_2_ - partial pressure of carbon dioxide. T1 – before the induction of anaesthesia, T2 – before cardiopulmonary bypass (CPB), T3 – 10 min after aortic cross-clamp (AoX), T4 – 10 min after aorta clamp release, T5 – 30 min after weaning from CPB, T6 – admission to the postoperative intensive care unit (PICU). Statistical significance was assessed using Dunnett’s post hoc test with T1 as the reference time point

Our analysis revealed a statistically significant increase in pH after induction of anaesthesia (before CPB) at the time point analysed (T2; *p* < 0.05; Fig. 1A) relative to the pH determined in serum before anaesthesia (T1). Normalisation of pH occurred temporarily within 10 min after aortic clamping (*p* > 0.05) and then decreased significantly at 10 min after clamp release (*p* < 0.001). Re-normalisation of pH occurred 30 min after disconnection from the CPB (*p* > 0.05) and then increased significantly on admission to the PICU (*p* < 0.001, Fig. 1A).

For pO_2_ changes, a statistically significant increase was observed after the induction of anaesthesia (before CPB) (T2, *p* < 0.001, Fig. 1B) relative to the serum level determined before anaesthesia (T1). Normalisation of pO_2_ levels did not occur, even on admission to the PICU (T6, *p* < 0.001).

When pCO_2_ changes were analysed (Fig. 1C), a statistically significant decrease in pCO_2_ was observed after anaesthesia (before CPB) (T2, *p* < 0.05) relative to serum pCO_2_ determined before anaesthesia (T1). Normalisation of pCO_2_ occurred temporarily at 10 min after aortic clamping and at 10 min after clamp release (*p* > 0.05). At 30 min after disconnection from CPB, pCO_2_ decreased statistically significantly, and its normalisation did not occur even on admission to the PICU (*p* < 0.001).

The nitrite/nitrate ratio increased significantly before CPB relative to pre-anaesthesia levels (*p* < 0.001; Fig. 1D). The observed increase occurred in 22 patients (69%), regardless of BMI or anaesthesia type. Among them, six hours after the surgical procedure, 19 patients (86%) showed increased leukocyte counts, 20 patients (91%) had increased CRP levels, and all patients showed increased troponin and creatinine levels. Moreover, the increase was also observed 10 min after aortic clamping (T3, *p* < 0.01), and the analysed ratio normalised quickly within 10 min after clamp release (T4, *p* > 0.05).

To better characterise the early pre-CPB physiological pattern observed at T2, we performed a correlation analysis between the nitrite/nitrate ratio and selected arterial blood gas parameters (Table 2).

**Table 2:** Spearman’s rank correlation coefficient between arterial blood gas parameters at time point T2 in patients undergoing CABG

	pH	nitrite/nitrate	pO₂	pCO₂
pH	X	0.08	**0.37**	**−0.68**
nitrite/nitrate	0.08	X	**0.46**	−0.19
pO₂	**0.37**	**0.46**	X	**−0.41**
pCO₂	**−0.68**	−0.19	**−0.41**	X

Correlations were assessed using the Spearman rank correlation test

Statistically significant correlations (*p* < 0.05) are highlighted

*pO*_*2*_ partial pressure of oxygen, *PpCO*_*2*_ partial pressure of carbon dioxide

Spearman rank correlation analysis revealed a strong, statistically significant inverse correlation between pH and pCO₂ (*r* = −0.68, *p* < 0.01), consistent with respiratory alkalosis. A moderate negative correlation was also observed between pCO₂ and pO₂ (*r* = −0.41, *p* < 0.05), suggesting a ventilation-related gas-exchange pattern. Furthermore, pO₂ showed a moderate positive correlation with both pH (*r* = 0.37, *p* < 0.05) and the nitrite/nitrate ratio (*r* = 0.46, *p* < 0.05), indicating that higher oxygenation was associated with elevated pH and changes in oxidative status. Correlations between the nitrite/nitrate ratio and pH or pCO₂ were not statistically significant.

### Analysis of Biochemical Parameters Revealing Early Perioperative Changes Within 10 min After Aortic Cross-clamp (AoX) (Time Point T3)

**Fig. 2:**
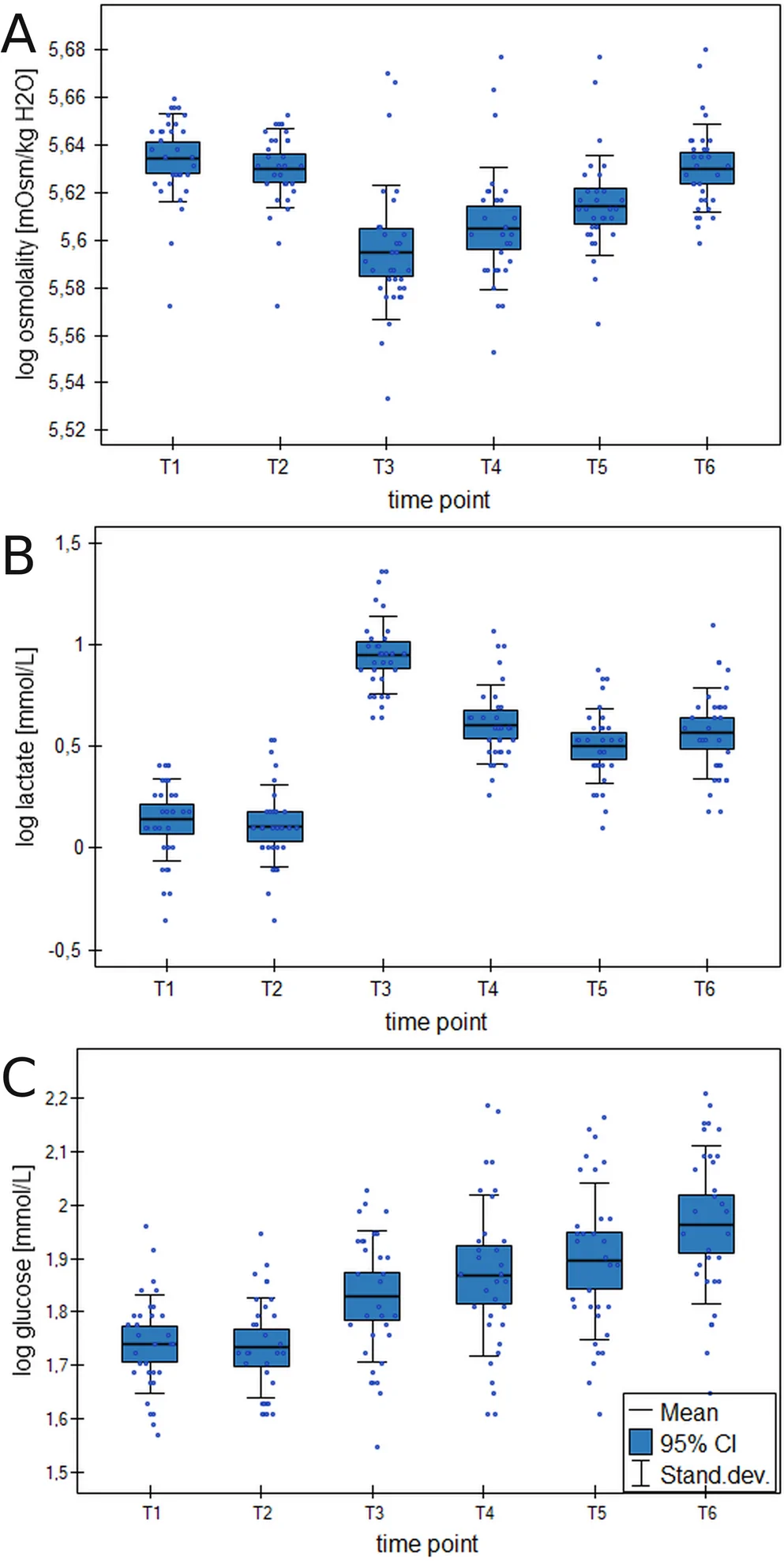
Analysis of biochemical parameters revealing early changes within 10 min after AoX (time point T3). Data are shown as mean values with 95% confidence intervals (95% CI) and standard deviation (Stand. dev.). T1 – before the induction of anaesthesia, T2 – before cardiopulmonary bypass (CPB), T3–10 min after aortic cross-clamp (AoX), T4–10 min after aorta clamp release, T5–30 min after weaning from CPB, T6 – admission to the postoperative intensive care unit (PICU). Statistical significance was assessed using Dunnett’s post hoc test with T1 as the reference time point

When osmolality changes were analysed, a statistically significant decrease was observed at 10 min after AoX (T3; *p* < 0.001) relative to serum osmolality levels measured before anaesthesia (T1). Normalisation of osmolality levels did not occur until admission to the PICU (T6; *p* > 0.05). Based on analysis of changes in lactate and glucose concentrations, a statistically significant increase in their levels was observed at 10 min after AoX (T3, *p* < 0.001) relative to pre-anaesthesia levels (T1; Fig. 2B-C). Normalisation of lactate and glucose levels did not occur, even on admission to the PICU (T6, *p* < 0.001). It should be noted that no association between the parameters determined was found at the analysed time point.

### Intraoperative Haemodynamic Profile

**Fig. 3:**
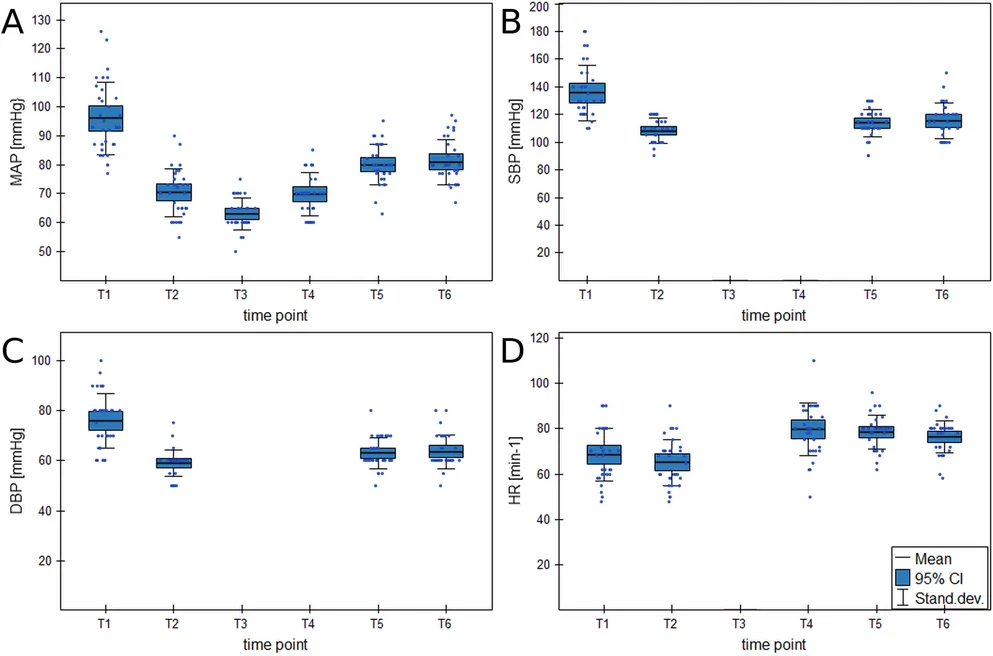
Intraoperative haemodynamic profile across predefined perioperative time points.Data are shown as mean values with 95% confidence intervals (95% CI) and standard deviation (Stand. dev.). MAP - mean arterial pressure; SBP – systolic blood pressure, DBP – diastolic blood pressure; HR – heart rate. T1 – before the induction of anaesthesia, T2 – before cardiopulmonary bypass (CPB), T3 – 10 min after aortic cross-clamp (AoX), T4 – 10 min after aorta clamp release, T5 – 30 min after weaning from CPB, T6 – admission to the postoperative intensive care unit (PICU).Statistical significance was assessed using Dunnett’s post hoc test with T1 as the reference time point.

Perioperative haemodynamic changes are presented in Fig. 3. Analysis of intraoperative haemodynamic parameters revealed significant time-dependent changes in MAP, SBP, DBP, and HR.

When MAP changes were analysed, a statistically significant decrease was observed at all subsequent perioperative time points relative to values measured before the induction of anaesthesia (T1). MAP was significantly lower before CPB (T2), 10 min after AoX (T3), 10 min after clamp release (T4), 30 min after weaning from CPB (T5), and on admission to the PICU (T6) (all *p* < 0.001, Dunnett’s post hoc test). The lowest MAP values were observed at T3, followed by a gradual increase at later time points. However, MAP remained significantly lower than T1 throughout the observation period, indicating incomplete haemodynamic recovery (Fig. 3A).

When SBP and DBP were analysed, a significant decrease was observed before CPB (T2) compared with T1 (*p* < 0.001). Although a partial increase occurred 30 min after weaning from CPB (T5) and on admission to the PICU (T6), both SBP and DBP remained significantly lower than T1 at these time points (all *p* < 0.001), indicating incomplete restoration of arterial blood pressure (Fig. 3B, C).

In contrast, heart rate (HR) demonstrated a distinct temporal profile. No statistically significant difference was observed before CPB (T2) compared with T1 (*p* > 0.05). However, HR increased significantly 10 min after clamp release (T4) and remained elevated at T5 and T6 (all *p* < 0.001 vs. T1). The highest HR values were observed at T4, followed by a slight decline at later time points; however, HR remained significantly higher after induction of anaesthesia, suggesting a persistent postoperative chronotropic response (Fig. 3D).

### Prediction of Early Kidney Dysfunction Based On 6-hour Postoperative Serum Creatinine Level

**Fig. 4:**
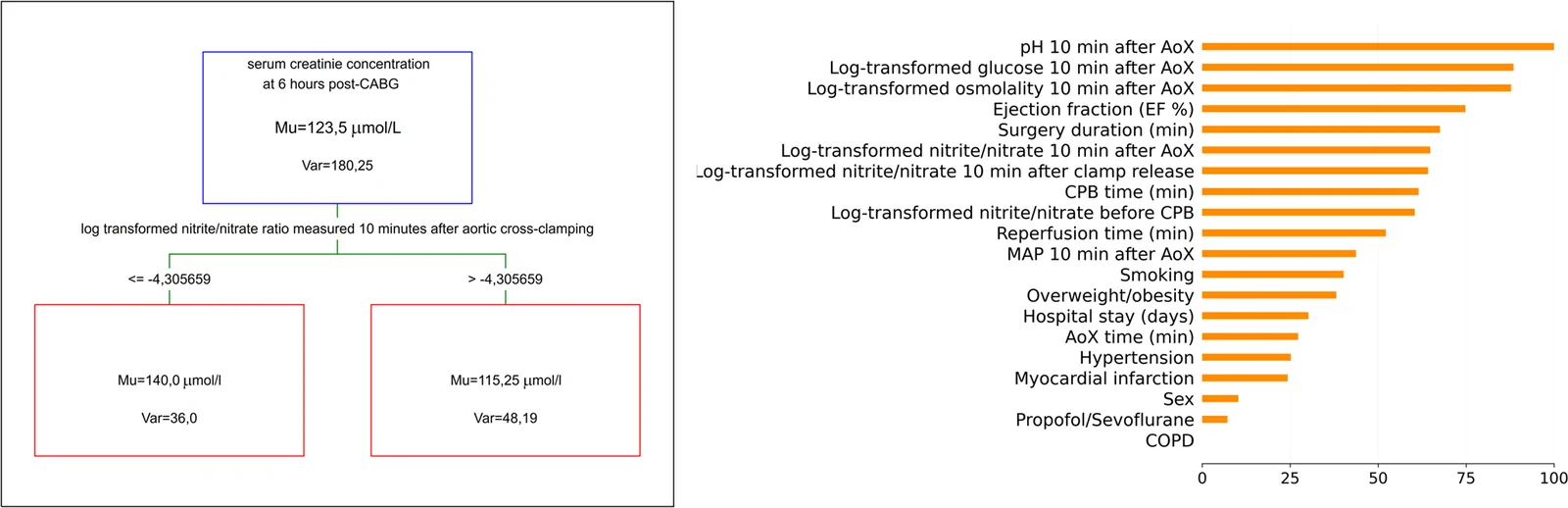
Predictor importance values in a regression tree model assessing early postoperative kidney dysfunction, with serum creatinine concentration measured at 6 hours post-CABG as the dependent variable. The model comprised one split node and two terminal nodes. Predictor importance reflects the relative contribution of each variable to the decision tree`s ability to reduce variance in the target outcome. Mu - mean value of the dependent variable within a given node; Var - the variance of the dependent variable within that node. AoX - aortic cross-clamp; CABG - coronary artery bypass grafting; COPD - chronic obstructive pulmonary disease; CPB - cardiopulmonary bypass; MAP - mean arterial pressure

A regression tree model was constructed to evaluate predictors of early postoperative kidney dysfunction, with serum creatinine concentration at 6 h post-CABG serving as the outcome variable. Among the analysed variables, the most influential predictors were: ejection fraction, reperfusion time, arterial blood pH measured 10 min after AoX, log-transformed nitrite/nitrate ratio and glucose concentration at the same time point. Other clinical variables, such as sex, BMI, COPD status, and length of hospital stay, demonstrated low predictive value (Fig. 4).

The decision tree identified the log-transformed nitrite/nitrate ratio measured 10 min after AoX as the primary split variable. Patients with a ratio ≤ − 4.31 had significantly higher postoperative creatinine levels (mean = 140 µmol/L), compared to those with a ratio > − 4.31 (mean = 115.25 µmol/L) (Fig. 4). This suggests that perioperative log-transformed nitrite/nitrate ratio measured 10 min after AoX may be associated with early postoperative renal marker increase.

The relatively high mean squared error in the test set (MSE = 845.63, with a standard error of ± 365.14) indicates variability in prediction accuracy, likely due to biological heterogeneity among samples.

### Prediction of Early Postoperative Inflammatory Marker Response Based On 6-hour Postoperative CRP Level

**Fig. 5:**
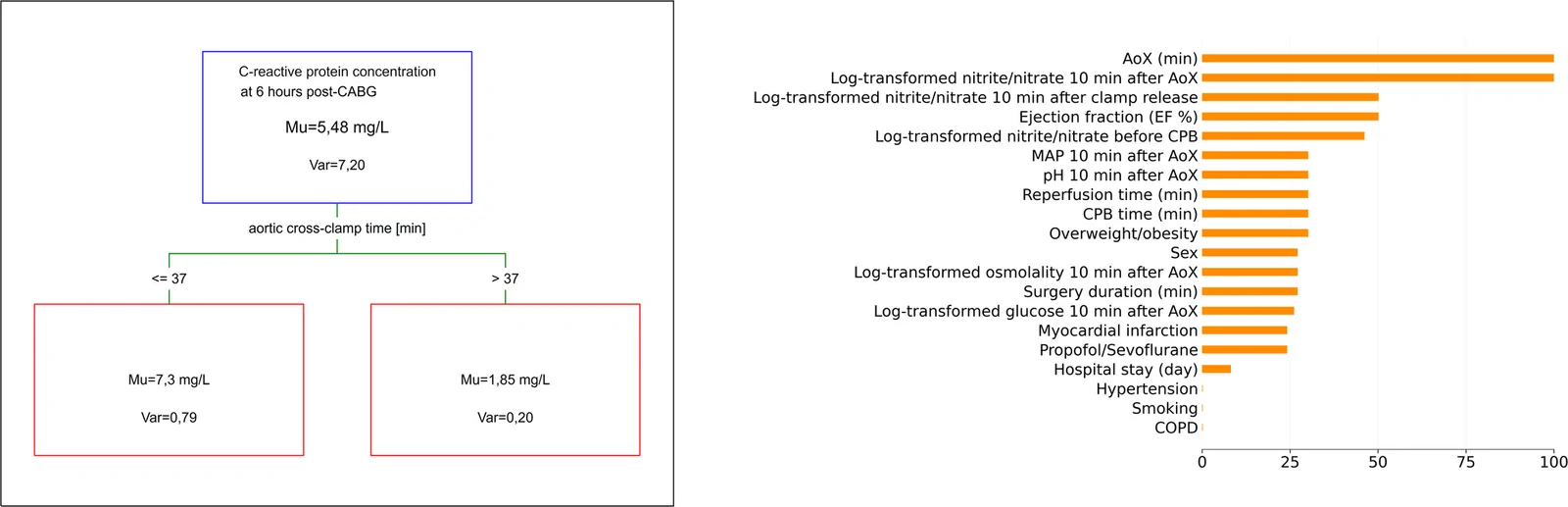
Predictor importance values in a regression tree model assessing early postoperative inflammatory response, with C-reactive protein (CRP) concentration measured at 6 hours post-CABG as the dependent variable. The model comprised one split node and two terminal nodes. Predictor importance reflects the relative contribution of each variable to the decision tree`s ability to reduce variance in the target outcome. Mu - mean value of the dependent variable within a given node; Var - variance of the dependent variable within that node. CABG - coronary artery bypass grafting; COPD - chronic obstructive pulmonary disease; CPB - cardiopulmonary bypass; MAP - mean arterial pressure; T2 – time point before CPB

A regression tree model was constructed to evaluate predictors of early postoperative systemic inflammation, with CRP concentration at 6 h post-CABG serving as the outcome variable. Among the analysed variables, the most influential predictors were: AoX time; log-transformed nitrite/nitrate ratio measured 10 min after cross-clamping and at the end of surgery; arterial blood pH measured 10 min after cross-clamping; and reperfusion time. Other clinical parameters, such as sex, smoking status, myocardial infarction, and hospital length of stay, demonstrated low predictive value (Fig. 5).

The decision tree identified AoX time as the primary split variable. Patients with AoX ≤ 37 min had significantly higher postoperative CRP levels (mean = 7.3 mg/L), compared to those with AoX > 37 min (mean = 1.85 mg/L) (Fig. 5). This finding suggests that shorter AoX times may be associated with a more pronounced early postoperative inflammatory marker.

The test-set error (MSE = 18.23 mg²/L², with a standard error of ± 11.22) should be interpreted cautiously given the small cohort size and the absence of external validation. Therefore, the tree analysis should be considered an exploratory approach to identify candidate variables associated with early postoperative CRP levels, rather than evidence of a generalizable prediction model.

## Discussion

The main finding of this exploratory observational cohort study is that high-BMI patients undergoing on-pump CABG exhibit dynamic perioperative changes in the plasma nitrite/nitrate ratio that coincide with acid-base, oxygenation, metabolic and haemodynamic shifts, and that these changes are associated with early postoperative creatinine and inflammatory marker levels. There is an additional risk of complications after cardiac surgery in obese and overweight patients. Therefore, studies analysing the most sensitive moments of anaesthesia procedures during complex surgeries, such as on-pump CABG surgery, may help improve care for overweight patients. This study aimed to evaluate perioperative changes in the plasma nitrite/nitrate ratio and selected biochemical and hemodynamic variables during CABG in patients with increased BMI. As the inflammatory process is induced in the perioperative period, analyses were performed at selected points relevant to the CABG procedure. The study used nitric oxide levels to indicate early postoperative inflammatory marker response.

During anaesthesia, significant changes in blood gas parameters were observed, including increased pH and pO₂, decreased pCO₂, and reduced osmolality. These shifts result primarily from mechanical ventilation, which elevates pO₂ and lowers pCO₂, leading to respiratory alkalosis. Further alterations result from the CPB initiation. Hyperoxia is common during CABG and may offer benefits such as reduced microemboli formation and stimulation of myocardial preconditioning. However, it can also contribute to adverse effects, including lung injury, increased production of reactive oxygen species, exacerbation of myocardial reperfusion injury, and reduced capillary density, leading to impaired tissue oxygenation. Therefore, perioperative hyperoxia should be used cautiously [13, 14]. Changes in plasma osmolality may result from the priming volume used during CPB. Besides isotonic crystalloids, the priming fluid includes hyperosmolar components, such as sodium bicarbonate (NaHCO₃) and 15% mannitol, which contribute to haemodilution and reduced colloid oncotic pressure [15]. Interpretation of the osmotic balance is further complicated by the administration of cardioplegic solution, which has an osmolality of 304 mOsm/L according to the manufacturer. Nitric oxide plays a key role in physiological and pathophysiological processes relevant to anaesthesiology and intensive care, including regulation of circulation, neural transmission, pain, immune response, metabolism, and mitochondrial function. Reduced nitric oxide bioavailability is associated with cardiovascular disease and metabolic syndrome, while its excess contributes to hypotension in septic shock [6, 12, 14, 16]. Direct measurement of nitric oxide in vivo is difficult, so its levels are estimated *via* nitrate and nitrite concentrations. Nitrate, the main oxidation product, is present in plasma at higher levels than nitrite, largely due to their different half-lives (5–6 h vs. ~20 min, respectively). Additionally, nitrate is continuously excreted by the kidneys [9–11]. In systemic inflammation (e.g., sepsis, severe gastroenteritis), nitrate and nitrite levels increase due to iNOS induction, whereas endothelial dysfunction (e.g., hypertension, diabetes, atherosclerosis) is associated with reduced NO levels [5, 8, 17]. Both obesity and cardiovascular diseases are prone to iNOS overexpression [18]. Nitrite, less influenced by diet, more accurately reflects local iNOS activity during inflammation [11] and, together with the nitrite/nitrate ratio, has been explored as a potential clinical biomarker, including for inflammatory complications such as those observed after COVID-19 [5].

This study showed that patients with elevated BMI undergoing CABG with CPB experienced significant perioperative changes in the nitrite/nitrate ratio and key biochemical parameters. Before CPB, a transient increase in the nitrite/nitrate ratio, pH, and pO₂, along with decreased pCO₂, was observed. At 10 min after aortic cross-clamping, lactate and glucose levels increased, while osmolality decreased. Normalisation of the nitrite/nitrate ratio occurred within 10 min after clamp release. Correlation analysis demonstrated a positive relationship between the nitrite/nitrate ratio, pO₂, and pH. The rapid normalisation of the nitrite/nitrate ratio may be clinically favourable. Earlier studies reported increases in nitrite during CPB and 10 min later [14, 19]. Pre-CPB nitrite elevation in our study may result from stress, mechanical ventilation, or heparin administration [20]. CPB itself does not appear to stimulate NO production in patients undergoing CABG, and peripheral sources, such as the lungs or muscle, may contribute [20]. Nitric oxide transport via S-nitrosohaemoglobin and its release during deoxygenation regulate microvascular flow, with nitrite bioactivation peaking during rapid deoxygenation, with maximum conversion and NO-mediated effects at 50% haemoglobin oxygen saturation [11, 21, 22]. Additionally, nitrite-induced vasodilation is enhanced under acidosis, which may explain the observed effects after cross-clamping and reperfusion (10 min after clamp release). Furthermore, transient tissue nitrosylation, accompanied by prolonged changes in blood gas parameters (pO2, pCO2) and osmolality, may positively influence serine/threonine kinase signalling, thereby preconditioning cardiac systolic function and improving cardiac performance after reperfusion in operated patients [23]. The systemic relationship between nitrite and nitrate is a complex issue that depends on many factors, including reduced capacity of the oral and intestinal microbiota [10]. Furthermore, fluctuations in their levels may influence the incidence of myocardial infarction after CABG surgery [24, 25]. Observed increases in CRP and WBC are typical of uncomplicated CABG procedures and reflect the inflammatory response. Despite CRP’s evident postoperative increase, it may also be responsible for postoperative atrial fibrillation, which seriously deteriorates patients’ haemodynamic status [26]. Postoperative troponin elevation is justifiable due to direct surgical manipulation of the myocardium, and the degree of elevation did not indicate underlying myocardial ischaemia. Unfortunately, a postoperative increase in creatinine may indicate early postoperative renal dysfunction Previous studies suggest that neither off-pump techniques nor shorter CPB duration fully protects against acute kidney injury (AKI), which may be linked to regional hypoxia and reduced nitric oxide (NO) availability due to endothelial dysfunction [27]. NO therapy has been shown to reduce AKI incidence and renal injury biomarkers, while obesity itself is a significant risk factor [28, 29]. The nitrite/nitrate ratio measured 10 min after aortic cross-clamping (AoX) may be associated with early postoperative renal dysfunction in high-BMI patients. However, perioperative concentrations of NO metabolites are influenced by oxygenation, ventilation, haemodilution, renal clearance, CPB-related changes in flow and shear stress, vasopressor requirements, inflammatory activation and reperfusion injury. Therefore, the observed associations most likely reflect an integrated perioperative NO-related response rather than a single causal pathway. Regression analysis identified AoX duration as the strongest predictor of postoperative inflammation, with shorter AoX times (≤ 37 min) paradoxically linked to higher CRP levels at 6 h post-CABG, suggesting intensified ischemia–reperfusion inflammatory responses. The observed association between shorter AoX duration and higher 6-hour postoperative CRP concentration is counterintuitive and should be interpreted with caution. This result may reflect sample variability, residual confounding, early timing of CRP measurement, or instability of the decision-tree split in a small dataset. CRP is a downstream acute-phase protein whose perioperative increase is largely mediated by cytokine activation, particularly IL-6; therefore, a single 6-hour postoperative CRP measurement may not fully capture the later inflammatory peak after cardiac surgery. Moreover, postoperative CRP concentration may be influenced by baseline inflammatory status, surgical tissue trauma, hemodilution, reperfusion-related oxidative stress, CPB-related immune activation and individual hepatic acute-phase responsiveness. Interindividual variability in CRP levels may also be partly genetically determined, as Brull et al. (2003) showed that polymorphisms within the human CRP gene influence circulating CRP concentrations [30]. Although CRP genotype was not assessed in the present study, this observation supports the possibility that early postoperative CRP values may vary between patients independently of AoX duration alone. It should therefore be considered a hypothesis-generating observation rather than evidence of a causal relationship between shorter cross-clamp duration and intensified inflammation. Variable importance analysis further highlighted the predictive role of the nitrite/nitrate ratio measured 10 min after AoX and after clamp release, pointing to NO-related oxidative stress in early inflammation. These results support the use of metabolic and perfusion-related biomarkers for early risk stratification in overweight and obese patients undergoing CPB, warranting further investigation into obesity-related inflammatory mechanisms and microcirculatory regulation. The haemodynamic profile provides important context for the observed biochemical changes. Sustained reductions in MAP, SBP, and DBP, along with a delayed increase in HR after clamp release and postoperatively, indicate persistent circulatory stress rather than rapid recovery. This pattern is consistent with cardiopulmonary bypass and reperfusion effects, including vasodilation, altered vascular tone, inflammation, and compensatory chronotropic response. Thus, changes in nitric oxide metabolites likely reflect broader disturbances in perfusion and vascular reactivity during and after CPB.

### Study Limitations

First, the study is limited by a small sample size, which reduces generalisability. The absence of a normal-BMI control group is another major limitation. Only eight normal-BMI patients were available from the original screened population, which was insufficient for reliable statistical comparison. Adding such a small control group would risk unstable estimates and potentially misleading conclusions. In addition, the original perioperative sampling protocol cannot be repeated under identical clinical, procedural and preanalytical conditions. Therefore, the present findings should be interpreted as exploratory observations limited to overweight and obese patients undergoing on-pump CABG. Another limitation is that anaesthetic technique was not randomised, and cumulative anaesthetic exposure was not analysed as a primary variable. Next, inflammation was assessed using CRP and leukocyte count, which, although routinely used in clinical practice, are non-specific; more detailed markers (e.g., cytokines, endothelial activation) would improve interpretation. The nitrite/nitrate ratio should not be used as an isolated biomarker of renal or inflammatory complications, as nitric oxide metabolites are influenced by multiple perioperative factors. Finally, decision tree findings are interpreted with caution due to the relatively small sample size and are considered exploratory. The tree-based analysis identified variables associated with early postoperative creatinine concentration rather than providing a validated prediction of clinically confirmed AKI. The study was not designed to validate a diagnostic biomarker and did not include sufficiently powered, independently defined clinical endpoints. Such analyses should be conducted in future prospective studies with adequate sample size, predefined AKI and inflammatory outcomes, and external validation.

## Conclusions

These findings suggest for the first time that perioperative NO-related metabolic changes may be associated with early postoperative renal and inflammatory marker responses in high-BMI patients undergoing on-pump CABG. The results are exploratory and hypothesis-generating and require validation in larger prospective cohorts with appropriate comparator groups and externally validated predictive models.

## Data Availability

The datasets generated and/or analysed during the current study are available in the MostWiedzy (DOI: [10.34808/5b61-0w08]), under CC BY licence.
